# Paradoxical regulation of Bcl-2 family proteins by 17β-oestradiol in human breast cancer cells MCF-7

**DOI:** 10.1038/sj.bjc.6690706

**Published:** 1999-10

**Authors:** L K Leung, T T Y Wang

**Affiliations:** Basic Research Laboratory, Division of Basic Sciences, National Cancer Institute, Building 560/12-05 NCI-FCRDC, Frederick, MD, 21702-1201, USA

**Keywords:** apoptosis, breast cancer, Bcl-2, Bcl-x(L), 17β-oestradiol

## Abstract

Tumorigenesis is related to the dysregulation of cell growth or cell death pathways. Hence, elucidation of the mechanisms involved in the modulation of pro- or anti-apoptotic proteins is important in furthering understanding of breast cancer aetiology and may aid in designing prevention and treatment strategies. In the present study, we examined the role of 17β-oestradiol on the regulation of apoptosis in the breast cancer cell line MCF-7. Using multi-probe RNAase protection assays, we found changes in the mRNA levels of several Bcl-2 family proteins upon treatment of MCF-7 cells with 17β-oestradiol. Unexpectedly, we found a paradoxical effects of 17β-oestradiol on two anti-apoptotic proteins Bcl-2 and Bcl-x. Treatment with 17β-oestradiol resulted in up-regulation of Bcl-2 mRNA and protein, but down-regulated Bcl-x(L) mRNA and protein. The effect of 17β-oestradiol on Bcl-x(L) occurred at concentration-dependent fashion. The effect was specific to 17β-oestradiol since other steroid hormones exert no effect on Bcl-x(L). Tamoxifen, an anti-oestrogen, blocked the down-regulation of Bcl-x(L) by 17β-oestradiol demonstrating this effect is oestrogen receptor-dependent. We speculate that different members of the Bcl-2 family proteins may be regulated through different pathway and these pathways may be modulated by 17β-oestradiol. © 1999 Cancer Research Campaign


*British Journal of Cancer* (1999) **81**(3), 387–392
© 1999 Cancer Research Campaign
Article no. bjoc.1999.0706

### Paradoxical regulation of Bcl-2 family proteins by 17β-oestradiol in human breast cancer cells MCF-7

**LK Leung and TTY Wang**

Basic Research Laboratory, Division of Basic Sciences, National Cancer Institute, Building 560/12-05 NCI-FCRDC, PO Box B, Frederick, MD 21702-1201, USA

**Summary** Tumorigenesis is related to the dysregulation of cell growth or cell death pathways. Hence, elucidation of the mechanisms involved in the modulation of pro- or anti-apoptotic proteins is important in furthering understanding of breast cancer aetiology and may aid in designing prevention and treatment strategies. In the present study, we examined the role of 17β-oestradiol on the regulation of apoptosis in the breast cancer cell line MCF-7. Using multi-probe RNAase protection assays, we found changes in the mRNA levels of several Bcl-2 family proteins upon treatment of MCF-7 cells with 17β-oestradiol. Unexpectedly, we found a paradoxical effects of 17β-oestradiol on two anti-apoptotic proteins Bcl-2 and Bcl-x. Treatment with 17β-oestradiol resulted in up-regulation of Bcl-2 mRNA and protein, but down-regulated Bcl-x(L) mRNA and protein. The effect of 17β-oestradiol on Bcl-x(L) occurred at concentration-dependent fashion. The effect was specific to 17β-oestradiol since other steroid hormones exert no effect on Bcl-x(L). Tamoxifen, an anti-oestrogen, blocked the down-regulation of Bcl-x(L) by 17β-oestradiol demonstrating this effect is oestrogen receptor-dependent. We speculate that different members of the Bcl-2 family proteins may be regulated through different pathway and these pathways may be modulated by 17β-oestradiol. © 1999 Cancer Research Campaign

**Keywords:** apoptosis; breast cancer; Bcl-2; Bcl-x(L); 17β-oestradiol

Apoptosis is a physiological process that is crucial to the growth and development of multicellular organisms. Its dysregulation has been linked to tumorigenesis (Mikulski, 1994; Wyllie, 1997). Several proteins have been identified to be components of the complex apoptosis machinery. Bcl-2, which is associated with the t(14;18) chromosomal breakpoint that occurs in follicular lymphoma, was the first protein identified to possess anti-apoptotic properties (Tsujimoto et al, 1985). Subsequently, additional proteins that share structural homology with Bcl-2 have been identified and characterized (Reed, 1994; McDonnell et al, 1996; Kroemer, 1997). These proteins, categorized as Bcl-2 family proteins, have been widely studied in programmed cell death and appear to possess either anti- or pro-apoptotic properties (Reed, 1994; McDonnell et al, 1996; Kroemer, 1997). In addition to the Bcl-2 family proteins, activation of the tumour necrosis factor (TNF) receptor family proteins by their respective ligands can trigger apoptosis (Wallach et al, 1997; Ashkenazi and Dixit, 1998). Initiation of apoptosis occur downstream of the ligand–receptor interaction through proteolytic cascade that involve caspases (Wallach et al, 1997; Ashkenazi and Dixit, 1998). Moreover, interaction between the two families of proteins mentioned above may also occur. It has been shown that anti-apoptotic proteins Bcl-2 and Bcl-x can inhibit various TNF receptor-mediated apoptotic events (Hermann et al, 1997; Srinivasan et al, 1998). Hence, coordination of various components of different apoptosis pathways may be necessary to ensure final execution of pro- or anti-apoptotic signals.

*Received 9 November 1998*
*Revised 25 March 1999*
*Accepted 20 April 1999*

*Correspondence to:* TTY Wang

Perturbation of Bcl-2 family proteins, and consequently apoptosis, may be important in mammary carcinogenesis. Overexpression of the long form of the Bcl-x protein has been observed in invasive breast cancer, and using Bcl-x protein expression as a prognostic tool for monitoring breast cancer progression has been suggested (Olopade et al, 1997). In addition, others have shown that expression of Bax in normal breast tissues is significantly higher than in malignant breast tissues (Bargou et al, 1995). Exposure to oestradiol has been found to be associated with increased risk in development of mammary tumour (Fishman et al, 1995). Given the involvement of Bcl-2 family proteins in tumorigenesis (Reed, 1994; McDonnell et al, 1996; Kroemer, 1997), modulation of the Bcl-2 family anti- or pro-apoptotic proteins by oestradiol may play a critical role in the mammary carcinogenesis. Recent work has indicated a potential role for 17β-oestradiol in modulation of Bcl-2 family proteins, such as the anti-apoptotic protein Bcl-2 (Wang and Phang, 1995; Huang et al, 1997) and pro-apoptotic protein Bak (Leung et al, 1998). These results appear to correlate with the anti-apoptotic property of oestradiol (Kyprianou et al, 1991). However, it remains unclear if various Bcl-2 family proteins may be coordinately regulated by oestradiol. Given that therapeutic and prevention strategies utilizing anti-oestrogens have been actively explored (Jordan, 1998), a better understanding of the role of oestradiol in modulating the Bcl-2 family protein-related apoptosis pathways may benefit development of breast cancer therapeutic and prevention strategies. To address the possible complex interaction of oestradiol with apoptosis pathways and taking into consideration that oestradiol, functioning through oestrogen receptor, can serve as transcriptional activator, we (1) examined the effects of oestradiol on Bcl-2 family proteins at both the message and protein levels and (2) asked whether various pro- or anti-apoptotic proteins can be regulated by oestradiol in a coordinated fashion. Our results indicate that (1) Bcl-2

family proteins may be differentially regulated by 17β-oestradiol and (2) 17β-oestradiol exert paradoxical effects on the anti-apoptotic proteins Bcl-2 and Bcl-x(L).

### MATERIALS AND METHODS

### Chemicals

Progesterone, tamoxifen, dihydrotestosterone and 17β-oestradiol were purchased from Sigma (St Louis, MO, USA). Recombinant human insulin-like growth factor-I (IGF-I) and recombinant human epidermal growth factor (EGF) were obtained from Promega (Madison, WI, USA). All other chemicals were from the best sources available.

### Cell culture

MCF-7 cells were cultured as previously described (Wang and Phang, 1995). Briefly, 1 week before initiation of the experiment, cells were switched to phenol red-free RPMI-1640 (Biofluids, Rockville, MD, USA) supplemented with 5% charcoal dextran-treated fetal bovine serum (CDS) (Hyclone Laboratories Inc., Logan, UT, USA), 2 mM glutamine, 100 units $ml^{-1}$ penicillin, 100 µg $ml^{-1}$ streptomycin, 1 M insulin and 2 ng $ml^{-1}$ hydrocortisone for 3 days. Subsequently, cells were switched to media without insulin and hydrocortisone, and 1 day before treatment cells were trypsinized and plated in phenol red-free RPMI-1640 containing 2 mM glutamine, 100 units $ml^{-1}$ penicillin, 100 µg $ml^{-1}$ streptomycin and 5% CDS.

### Determination of cell number and apoptosis for MCF-7 cells

We used DNA fragmentation as the criteria for apoptotic cell death. DNA fragmentation was measured using the cell death enzyme-linked immunosorbent assay (ELISA) (Boehringer, Indianapolis, IN, USA). Cells ($5 \times 10^4$ cell) were plated in each well of 24-well plates. After treatment, the cells were washed once with phosphate-buffered saline and 0.5 ml lysis buffer was added. After a 30-min incubation the supernatant was recovered and assayed for DNA fragments according to manufacturer's protocol. Additional plates identically treated as above were analysed for cell number using the sulphorhodamine assay (Wang and Phang, 1995). The OD 405 obtained from the DNA fragmentation assay was then normalized for cell number and the results are expressed relative to untreated control.

### Total RNA isolation, multi-probe RNAase protection assay and RT-PCR

MCF-7 cells were grown in 6-well Costar plates ($2 \times 10^6$ cells $well^{-1}$), and total RNA was isolated as described previously (Wang and Phang, 1995). Multi-probe RNAase protection assay for Bcl-2 family and TNF receptor family protein were performed using the RiboQuant RNase protection assay (Pharmingen, San Diego, CA, USA) according to the manufacturer's protocol. The template set used were hAPO-2 for Bcl-2 family proteins. The protected fragments were visualized by autoradiography and quantitated using a phosphoimager (BioRad GS-360) respectively. Phosphoimager readings were normalized for glyceraldehyde 3-phosphate dehydrogenase (GAPDH) content. Determination of Bcl-x mRNA levels using semiquantitative reverse transcription polymerase chain reaction (RT-PCR) were performed as follows. First-strand synthesis was performed using a RT-PCR kit from Stratagene (CA, USA) using 0.1–0.5 µg of total RNA. Subsequent cDNA amplification was performed using the following primers for Bcl-x: 5′-AAT GTC TCA GAG CAA CCG GGA GCT G-3′ (forward primer) and 5′-TCA TTT CCT ACT GAA GAG TGA GCC CA-3′ (reverse primer), primers for GAPDH were obtained from Clontech (CA, USA). The conditions for PCR were as previously described (Wang and Phang, 1995) except 20 cycles were performed in the presence of 10 µCi of [α-$P^{32}$] dATP (3000 Ci $mmol^{-1}$) per reaction. The linearity of the amplification cycles was confirmed by separate experiments. The PCR products were then separated on a 2% agarose gel. The gel was dried and exposed to a Kodak X-OMAT AR film or phosphoimager screen (BioRad, Richmond, CA, USA). The amplified cDNA fragments were visualized by autoradiography and quantitated using a phosphoimager (BioRad GS-360) respectively. Phosphoimager readings were normalized for GAPDH content. The Bcl-x primers were designed to amplify both long (702 bp) and short forms (488 bp), but the short form was not detectable under the conditions used.

### Immunodetection of Bcl-2 family proteins levels

MCF-7 cells were plated in 100-mm dishes at $5 \times 10^6$ cells $dish^{-1}$ and treatments were begun 24 h after plating. Treated cells were harvested by washing once with phosphate-buffered saline (PBS), pH 7.4, and scraping from the dish into 0.5 ml buffer (PBS, pH 7.4, 1% NP40, 0.5% sodium deoxycholate, 0.1% sodium dodecyl sulphate (SDS) supplemented with protease inhibitors (100 µg $ml^{-1}$ phenylmethyl sulphonyl fluoride, 1 µg $ml^{-1}$ aprotinin, 1 µg $ml^{-1}$ leupeptin, 1 mM EDTA, 1 µg $ml^{-1}$ pepstatin). Cells were then lysed by sonication on ice (Tekmar Sonic Disruptor, 30% power, 30 s) to obtain cell lysate for Western blotting. Protein concentration of the cell lysate was determined by the Dc protein assay (Bio-Rad, Richmond, CA, USA). Aliquots of cell lysate containing 15–25 µg of protein were separated on 10% SDS polyacrylamide gel electrophoresis (SDS-PAGE) (Novex, San Diego, CA, USA) and electro-transferred to polyvinyldifluoride membrane (Millipore, Bedford, MA, USA). The membranes were then probed with antibody against Bcl-2 (Dako, Carpinteria, CA, USA), Bcl-x (Santa Cruz Biotechnology Inc., Santa Cruz, CA, USA), Bak (Upstate Biochemicals, Lake Placid, NY, USA) or Bax (Santa Cruz Biotechnology Inc., Santa Cruz, CA, USA) and visualized with the enhanced chemiluminescence method (Pierce, IL, USA).

### RESULTS

### Effects of 17β-oestradiol on cell number and apoptosis in human breast cancer cell line MCF-7

Treatment of oestrogen receptor (ER)-positive MCF-7 cell with oestradiol can result in increased cell numbers. As shown in Figure 1A, MCF-7 cell cultured in presence of $10^{-10}$ M 17β-oestradiol for 48 h resulted in significantly higher cell number than the untreated control. This effect of 17β-oestradiol on cell number homeostasis appeared exerted in part through a decrease in apoptosis. The relative apoptotic index for cells cultured in presence of oestradiol were significantly lower than cells cultured in absence of 17β-oestradiol (Figure 1B).

 *© 1999 Cancer Research Campaign*

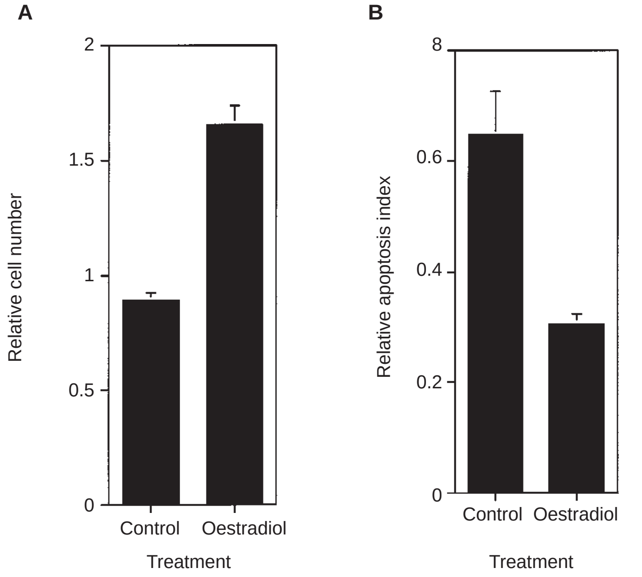

**Figure 1** Effects of 17β-oestradiol on cell number homeostasis. MCF-7 cells were cultured in the presence or absence of 17β-oestradiol ($10^{-10}$ M) for 48 h. Cell number and apoptosis index determined as described in Materials and Methods. (**A**) Comparison of cell number. (**B**) Comparison of apoptosis index

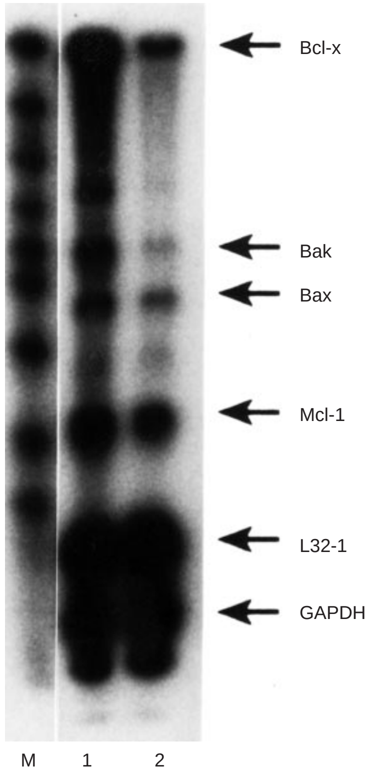

**Figure 2** Effect of 17β-oestradiol on the mRNA levels of Bcl-2 family proteins assessed using RNAase protection assay. MCF-7 cells were cultured in the presence or absence of 17β-oestradiol ($10^{-10}$ M) for 48 h, RNA isolated and multi-probe RNAase protection assay performed as described in Materials and Methods. Lane 1, control. Lane 2, oestradiol. M: untreated probes

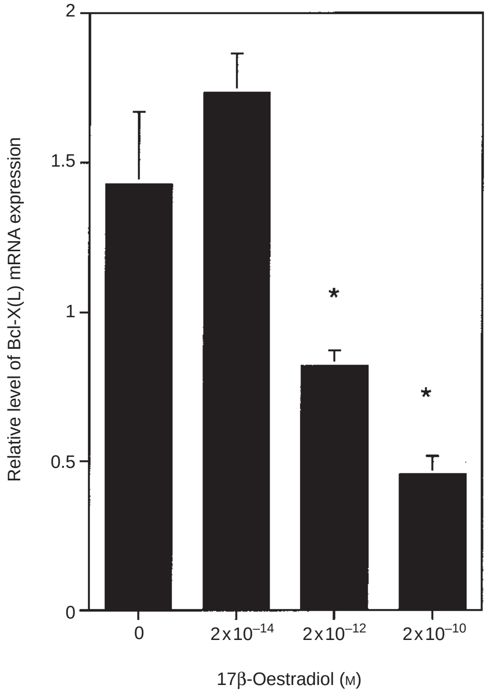

**Figure 3** Concentration-dependent effects of 17β-oestradiol on Bcl-x(L) mRNA levels. MCF-7 cells were treated with the indicated concentrations of 17β-oestradiol for 120 h. Total RNA was isolated, and the Bcl-x(L) mRNA level was determined as described in Materials and Methods. *Represents value (from three separate treatments) significantly different from that of control ($P < 0.05$, ANOVA)

### Effects of 17β-oestradiol on Bcl-2 family proteins' mRNA expression

To further understand the mechanism underlying the effect of oestradiol on apoptosis, we began by examining the effect of 17β-oestradiol on the Bcl-2 family proteins, essential components of apoptosis pathways. Given that 17β-oestradiol can exert its effect through interaction with ER and serve as transcriptional factor, we initially examined the effects of oestradiol on the mRNA levels of the Bcl-2 family proteins. Taking advantage of a multi-probe RNAase protection assay, we compared mRNA expression of Bcl-2 family proteins in cells cultured in presence and absence of 17β-oestradiol (Figure 2). After normalization to GAPDH, we found several differences between cells cultured in the presence or absence of 17β-oestradiol. Unexpectedly we found treatment of cells with 17β-oestradiol led to a decrease in the anti-apoptotic protein Bcl-x mRNA levels (Figure 2A). Treatment with 17β-oestradiol also leads to alteration in another Bcl-2 family proteins, Bak, which we have previously reported (Leung et al, 1998).

### Concentration-dependent effects of 17β-oestradiol on Bcl-x mRNA expression

Down-regulation of the anti-apoptotic protein Bcl-x mRNA, as demonstrated above using multi-probe RNAase protection assay, appears to contradict the anti-apoptotic property of oestradiol. Therefore, to further confirm that treatment with 17β-oestradiol indeed led to decreased levels of Bcl-x mRNA, we used a semi-quantitative RT-PCR as an alternative method to assess this

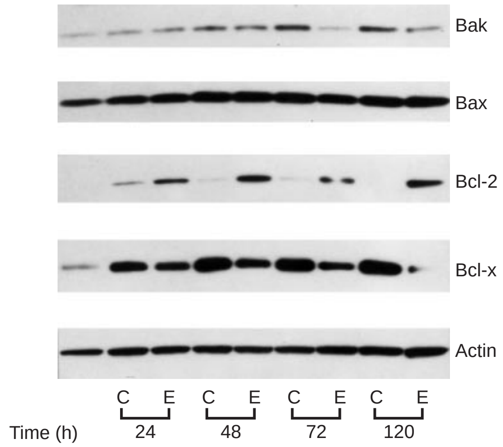

**Figure 4** Time-dependent effects of 17β-oestradiol on Bcl-2 family proteins. MCF-7 cells were cultured in the presence and absence of 17β-oestradiol ($10^{-10}$ M) as described in Materials and Methods. Cell were harvested at the indicated times and Bcl-2 family proteins determined by immunodetection as described in Materials and Methods. C: control, E: 17β-oestradiol

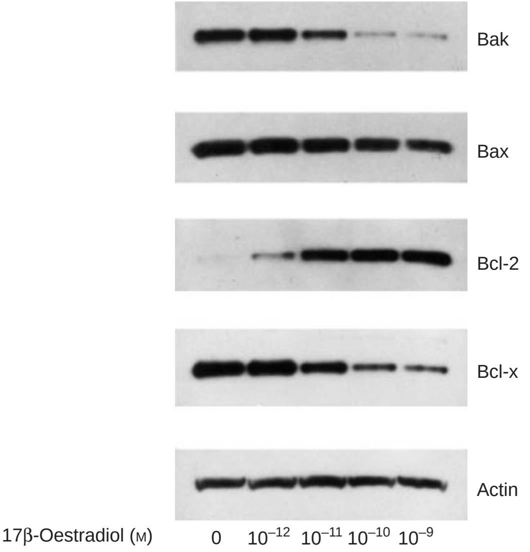

**Figure 5** Concentration-dependent effects of 17β-oestradiol on Bcl-2 family proteins. MCF-7 cells were cultured in the presence of varied concentrations of 17β-oestradiol as described in Materials and Methods. Cells were harvested at the indicated times and Bcl-2 family proteins determined by immunodetection as described in Materials and Methods

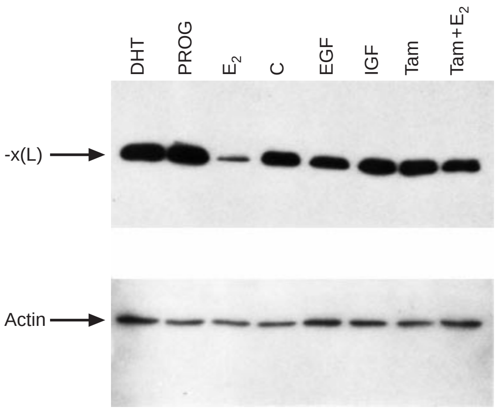

**Figure 6** Effects of various steroid hormones and growth factors on Bcl-x(L) protein levels. MCF-7 cells were cultured in 5% CDS with additional supplements of various steroid hormones or growth factors. Bcl-x(L) protein levels were determined as described in Materials and Methods. The immunoblot represents one of three separate experiments. The lanes are: (1) DHT, $10^{-9}$ M dihydrotestosterone; (2) PROG, $10^{-9}$ M progesterone; (3) C, control; (4) E, $10^{-10}$ M 17β-oestradiol; (5) EGF, 10 ng $ml^{-1}$ epidermal growth factor; (6) IGF, 10 ng $ml^{-1}$ insulin-like growth factor-I; (7) Tam, $10^{-6}$ M tamoxifen; (8) Tam+E, $10^{-6}$ M tamoxifen plus $10^{-10}$ M 17β-oestradiol. Upper panel: Bcl-x(L); lower panel: actin alteration in Bcl-x message. Consistent with the multi-probe RNAase protection assay, Bcl-x mRNA as assessed by semi-quantitative RT-PCR was significantly less in cells cultured in the presence of 17β-oestradiol than in untreated controls (Figure 3). In addition, the effect of 17β-oestradiol on Bcl-x mRNA levels appeared to be concentration-dependent. Under these RT-PCR conditions only the long form-Bcl-x(L), but not the short form-Bcl-x(S) of Bcl-x, mRNA were detected.

### Comparison of the effects of 17β-oestradiol on Bcl-x(L) to other Bcl-2 family proteins

Having established an effect of oestradiol on Bcl-x(L) mRNA, we further compared the effects of 17β-oestradiol on Bcl-x to several other Bcl-2 family proteins at the protein levels using immuno-detection. As illustrated in Figure 4, Bcl-x(L) protein increased in a time-dependent fashion. Treatment with 17β-oestradiol suppressed the time-dependent increase in Bcl-x protein, and the effects can be observed after 24 h. In contrast, treatment of MCF-7 cells with $10^{-10}$ M 17β-oestradiol resulted in a time-dependent increase in expression of the anti-apoptotic protein Bcl-2. The effects of 17β-oestradiol on Bcl-2 can also be seen after 24-h treatment. Treatment with 17β-oestradiol resulted in suppression of the increase in the pro-apoptotic protein Bak. The time course for oestradiol's effect on Bak appeared to be different from that of Bcl-2 or Bcl-x. Significant changes in Bak expression were detected only after 72 h treatment with 17β-oestradiol. In addition, we also compared the concentration effects of 17β-oestradiol on the Bcl-2 family proteins described above. As shown in Figure 5, the threshold of responses of anti-apoptotic protein Bcl-2, Bcl-x and pro-apoptotic proteins Bak, to 17β-oestradiol appeared to be similar. Significant changes in all three proteins can be observed at $10^{-11}$ M 17β-oestradiol. There were no significant differences in Bax protein at any time point or treatment.

### Specificity and mechanism of 17β-oestradiol's effect on Bcl-x(L) in MCF-7 cells

As we observed a difference in Bcl-x(L) levels in cells cultured in presence and absence of 17β-oestradiol, we asked whether this change was unique to 17β-oestradiol and whether this is through ER-dependent pathway. The effects of oestradiol on Bcl-x(L) was compared to that of various steroid hormones or growth factors with known biological effects on mammary cells. These factors included: 17β-oestradiol ($10^{-10}$ M), progesterone ($10^{-9}$ M), dihydro-testosterone ($10^{-9}$ M), EGF (10 ng $ml^{-1}$), or IGF-I (10 ng $ml^{-1}$). As illustrated in Figure 6, after a 5-day incubation, only treatment with 17β-oestradiol resulted in the suppression of Bcl-x(L), while other treatments did not alter Bcl-x(L) protein levels. The inhibitory effects of 17β-oestradiol appeared to be executed

*© 1999 Cancer Research Campaign*

through ER-dependent pathways. Treatment of cells with the anti-oestrogen tamoxifen ($10^{-6}$ M) abolished the inhibitory effect of 17β-oestradiol on Bcl-x(L) expression (Figure 6).

### DISCUSSION

Oestradiol is a known risk factor in the development of mammary cancer (Fisherman et al, 1995); however, the mechanism by which oestradiol exerts its effect is still unclear. Given the importance of apoptosis in tumorigenesis (Mikulski, 1994; Wyllie, 1997) and recent works implicating alteration of apoptosis pathways in mammary tumorigenesis (Bargou et al, 1995; Olopade et al, 1997), modulation of apoptosis pathways by oestradiol can be an important mechanism by which oestradiol can exert its effect on mammary carcinogenesis. It has been established, by this laboratory and others (Teixeria et al, 1995; Wang and Phang, 1995), that oestradiol induces proliferation of human mammary tumour cells in vitro, and this effect is due in part to a decrease in apoptosis. Figure 1 confirms that treatment of oestradiol can result in both increased cell numbers and decreased apoptosis. To further understand the mechanisms underlying the effect of oestradiol on apoptosis, it would be important to examine the effect of 17β-oestradiol on the various components of apoptosis pathways. With respect to components of apoptosis pathways much attention has been focused on Bcl-2, which has been shown to have potent anti-apoptotic effects on tumour cells (Reed, 1994; McDonnell et al, 1996; Kroemer, 1997). However, Bcl-2 is just one protein in a complex and diverse family of proteins which have been shown to have either anti- or pro-apoptotic properties (Reed, 1994; McDonnell et al, 1996; Kroemer, 1997). In addition, apoptosis has also been shown to be regulated by another family of proteins, the TNF receptor family (Wallach et al, 1997; Ashkenazi and Dixit, 1998). The regulation of these separate pathways and how alterations in expression of the various family members result in cell death is complicated and remains largely unclear. To elucidate the roles of oestradiol we began by investigating the effects of oestradiol on the mRNA levels of the Bcl-2 family proteins.

Interestingly, in an initial screening using a multi-probe RNAase protection assay (Figure 3) we observed that cells cultured in presence of 17β-oestradiol expressed lower levels of the anti-apoptotic protein Bcl-x mRNA than the untreated controls. Since Bcl-x has been shown to be anti-apoptotic (Hu et al, 1998; Srinivasan et al, 1998), down-regulation of Bcl-x(L) is at odds with the anti-apoptotic effect of oestradiol. Therefore, we further investigated the effect of oestradiol on Bcl-x. As shown in Figure 3, this effect of 17β-oestradiol on Bcl-x mRNA was subsequently confirmed by semi-quantitative RT-PCR and we found that treatment with 17β-oestradiol resulted in concentration-dependent changes in Bcl-x(L) mRNA. The changes in Bcl-x(L) mRNA were reflected at the protein level. As shown in Figures 4 and 5, treatment with 17β-oestradiol also resulted in lower levels of Bcl-x(L) protein. The effect of 17β-oestradiol appeared to be suppression of a temporal increase of Bcl-x(L) during culturing of MCF-7 cells. The level of Bcl-x(L) increased during the culturing periods (0–120 h), and the addition of 17β-oestradiol attenuated that increase. The effect of 17β-oestradiol on Bcl-x(L) is in contrast to the effects of 17β-oestradiol on another anti-apoptotic protein, Bcl-2. Treatment with 17β-oestradiol resulted in an increase in both Bcl-2 mRNA and protein levels. Treatment with 17β-oestradiol also affected the pro-apoptotic proteins Bak and Bax differently. Treatment with 17β-oestradiol resulted in down-regulation of Bak, but exerted little effect on Bax, both at the mRNA and protein levels. The concentration of 17β-oestradiol that elicited an effect on the various Bcl-2 family proteins was similar. However, there appeared to be a difference in temporal changes of various Bcl-2 family proteins upon exposure to 17β-oestradiol. While changes in Bcl-2 and Bcl-x(L) occurred as early as 24 h, the effect on Bak appeared to occur after 72 h.

In order to demonstrate the specificity of 17β-oestradiol as well as the mechanism of action, we compared the effects of oestradiol to other steroid hormones and cytokines. The addition of progesterone and dihydrotestosterone did not alter Bcl-x(L) levels. Thus, the effects of 17β-oestradiol on Bcl-x(L) appeared to be specific among the steroid hormones. In addition, oestradiol is known to affect growth factors, which are believed to be responsible for part of the proliferative responses induced by oestradiol (de Cupis and Favoni, 1997). Treatment with EGF or IGF-1 did not cause any changes in Bcl-x(L) levels. Thus, oestradiol does not appear to exert its effect on Bcl-x(L) indirectly through regulation of these growth factors. These results on Bcl-x is similar to what we have found for Bak (Leung et al, 1998) and Bcl-2 (TT Wang, unpublished observations). To determine whether the ER was necessary for oestradiol to exert its effect on Bcl-x(L), we treated cells with the anti-oestrogen tamoxifen. As shown in Figure 6, addition of tamoxifen negated the suppressive effects exerted by 17β-oestradiol, supporting that the effect of 17β-oestradiol was mediated through ER-dependent pathways. The effects of 17β-oestradiol on the Bcl-2 family proteins may be indirect, there is no apparent consensus of an oestrogen-responsive element in the Bcl-x or the Bcl-2 promoter sequences (Grillot et al, 1997). However, several other consensus sequences for various other transcriptional factors are present. Oestradiol may thus exert its effect indirectly through one or more of these pathways.

The present study and our previous work (Wang and Phang, 1995; Leung et al, 1998) clearly demonstrated that oestradiol coordinately regulated the expression of several within the Bcl-2 family of proteins, and this occurs in an ER-dependent manner. This supports our hypothesis that oestradiol may regulate apoptosis through its effect on Bcl-2 family proteins. However, since oestradiol inhibited apoptosis (Figure 1) and Bcl-x is known to possess anti-apoptotic property, the down-regulation of Bcl-x by oestradiol is difficult to interpret. However, we speculate that up-regulation of Bcl-x(L) may be associated with cell survival in the absence of proliferative signals. Up-regulation of Bcl-x(L) in absence of proliferative signal may serve to insure cell survival in the absence of growth signals. In the presence of oestradiol, however, the survival pathway may have been inactivated, thus down-regulation of Bcl-x occurred. By contrast, the other anti-apoptotic protein Bcl-2 may be associated with proliferative responses so that the cells can prevent apoptosis and take full advantages of proliferative signals induced by oestradiol. It is interesting, in this light, to consider a recent study which has shown that Bcl-x(L) is often over-expressed in ER-negative invasive tumours (Olopade et al, 1997). This is consistent with the current data, which show that Bcl-x(L) expression is higher in absence of an oestradiol signal.

In summary, 17β-oestradiol exerted a differential effect on Bcl-2 family proteins in ER-positive human breast cancer cell line MCF-7. Interestingly, 17β-oestradiol appeared to exert a paradoxical effect on the anti-apoptotic protein Bcl-2 and Bcl-x(L). We proposed that different pathways may be involved in regulation of Bcl-2 family proteins, and 17β-oestradiol may exert an effect on these pathways.

*© 1999 Cancer Research Campaign*

### ACKNOWLEDGEMENTS

The authors would like to thank Drs Henry P Ciolino and Susan N Perkins for suggestions on this manuscript.

### REFERENCES

Ashkenazi A and Dixit VM (1998) Death receptors: signaling and modulation. *Science* **281**: 1305–1308

Bardelli A, Longati P, Albero D, Goruppi S, Schneider C, Ponzetto C and Comoglio PM (1996) HGF receptor associates with the anti-apoptotic protein BAG-1 and prevents cell death. *EMBO J* **15**: 6205–6212

Bursch W, Ellinger A, Kienzl H, Torok L, Pandey S, Sirkorska M, Walker R and Hermann RS (1996) Active cell death induced by the anti-estrogens tamoxifen and ICI 164384 in human mammary carcinoma cells (MCF-7) in culture: the role of autophagy. *Carcinogenesis* **17**: 1595–1607

Bargou RC, Daniel PT, Mapara MY, Bommert K, Wagener C, Kallinich B, Royer HD and Dorken B (1995) Expression of the bcl-2 gene family in normal and malignant breast tissue: low bax-alpha expression in tumor cells correlates with resistance towards apoptosis. *Int J Cancer* **16**: 854–859

Chittenden T, Flemington C, Houghton AB, Ebb RG, Gallo GJ, Elangovan B, Chinnadurai G and Lutz RJ (1995) A conserved domain in Bak, distinct from BH1 and BH2, mediates cell death and protein binding functions. *EMBO J* **14**: 5589–5596

de Cupis A and Favoni RE (1997) Oestrogen/growth factor cross-talk in breast carcinoma: a specific target for novel antioestrogens. *Trends Pharmacol Sci* **18**: 245–251

Farrow SN, White JHM, Martinou I, Raven T, Pun K-T, Grinham CJ, Martinour J-C and Brown R (1995) Cloning of a bcl-2 homologue by interaction with adenovirus E1B 19K. *Nature* **374**: 731–73

Fishman J, Osborne MP and Telang NT (1995) The role of estrogen in mammary carcinogenesis. *Ann NY Acad Sci* **768**: 91–100

Grillot DA, Gonzalez-Garcia M, Ekhterae D, Duan L, Inohara N, Ohta S, Seldin MF and Nunez G (1997) Genomic organization, promoter region analysis, and chromosome localization of the mouse bcl-x gene. *J Immunol* **158**: 4750–4757

Herrmann JL, Beham AW, Sarkiss M, Chiao PJ, Rands MT, Bruckheimer EM, Brisbay S and McDonnell TJ (1997) Bcl-2 suppresses apoptosis resulting from disruption of the NF-kappa B survival pathway. *Exp Cell Res* **237**: 101–109

Huang Y, Ray S, Reed JC, Ibrado AM, Tang C, Nawabi A and Bhalla K (1997) Estrogen increases intracellular p26 Bcl-2 to p21 Bax ratios and inhibits taxol-induced apoptosis of human breast cancer MCF-7 cells. *Breast Cancer Res Treat* **42**: 73–81

Hu Y, Benedict MA, Wu D, Inohara N and Nunez G (1998) Bcl-XL interacts with Apaf-1 and inhibits Apaf-1-dependent caspase-9 activation. *Proc Natl Acad Sci USA* **95**: 4386–4391

Jordan VC (1998) Antiestrogenic action of raloxifene and tamoxifen: today and tomorrow. *J Natl Cancer Inst* **90**: 967–971

Kandouz M, Siromachkova M, Jacob D, Chretien-Marquet B, Therwath A and Gompel A (1996) Antagonism between estradiol and progestin on Bcl-2 expression in breast-cancer cells. *Int J Cancer* **68**: 120–125

Kluck RM, Bossy-Wetzel E, Green DR and Newmeyer DD (1997) The release of cytochrome c from mitochondria blocked. *Science* **275**: 1129–1132

Kroemer G (1997) The proto-oncogene Bcl-2 and its role in regulating apoptosis. *Nat Med* **3**: 614–620

Kyprianou N, English HF, Davidson NE and Isaacs JT (1991) Programmed cell death during regression of the MCF-7 human breast cancer following estrogen ablation. *Cancer Res* **51**: 162–166

Leung LK, Do L and Wang TTY (1998) Regulation of death promoter Bak expression by cell density and 17β-estradiol in MCF-7 cells. *Cancer Lett* **124**: 47–52

McDonnell TJ, Beham A, Sarkiss M, Andersen M and Lo P (1996) Importance of the Bcl-2 family in cell death regulation. *Experientia* **52**: 1008–1017

Mikulski SM (1994) Pathogenesis of cancer in view of mutually opposing apoptotic and anti-apoptotic growth signals (Review) *Int J Oncol* **4**: 1257–1263

Nenci I, Marchetti E and Queerzoli P (1988) Commentary on human mammary preneoplasia. The estrogen receptor-promotion hypothesis. *J Steroid Biochem* **30**: 105–106

Olopade OI, Adeyanju MO, Safa AR, Hagos F, Mick R, Thompson CB and Recant WM (1997) Over-expression of BCL-x protein in primary breast cancer is associated with high tumor grade and nodal metastases. *Cancer J Sci Am* **3**: 230–237

Pecci A, Scholz A, Pelster D and Beato M (1997) Progestins prevent apoptosis in a rat endometrial cell line and increase the ratio of bcl-XL to bcl-XS. *J Biol Chem* **272**: 11791–11798

Reed JC (1994) Bcl-2 and the regulation of programmed cell death. *J Cell Biol* **124**: 1–6

Saeed B, Zhang H and Ng SC (1997) Apoptotic program is initiated but not completed in LNCap cells in response to growth in charcoal-stripped media. *Prostate* **31**: 145–152

Sattle M, Liang H, Nettesheim D, Meadows RP, Harlan JE, Eberstadt M, Yoon HS, Shuker SB, Chang BS, Minn AJ, Thompson CB and Fesik SW (1997) Structure of Bcl-x(L)-Bak peptide complex: recognition between regulators of apoptosis. *Science* **275**: 983–986

Srinivasan A, Li F, Wong A, Kodandapani L, Smidt R Jr, Krebs JF, Fritz LC, Wu JC and Tomaselli KJ (1998) Bcl-xL functions downstream of caspase-8 to inhibit Fas- and tumor necrosis factor receptor 1-induced apoptosis of MCF7 breast carcinoma cells. *J Biol Chem* **273**: 4523–4529

Teixeira C, Reed JC and Pratt MA (1995) Estrogen promotes chemotherapeutic drug resistance by a mechanism involving Bcl-2 proto-oncogene expression in human breast cancer cells. *Cancer Res* **55**: 3902–3907

Tsujimoto Y, Cossman J, Jaffe E and Croce CM (1985) Involvement of the bcl-2 gene in human follicular lymphoma. *Science* **228**: 1440–1443

Wallach D, Boldin M, Varfolomeev E, Beyaert R, Vandenabeele P and Fiers W (1997) Cell death induction by receptors of the TNF family: towards a molecular understanding. *FEBS Lett* **410**: 96–106

Wang TT and Phang JM (1995) Effects of estrogen on apoptotic pathways in human breast cancer cell line MCF-7. *Cancer Res* **55**: 2487–2489

Wyllie AH (1997) Apoptosis and carcinogenesis. *Eur J Cell Biol* **73**: 189–197

Yang J, Liu X, Bhalla K, Kim CN, Ibrado AM, Cai J, Peng T-I, Jones DP and Wang X (1997) Prevention of apoptosis by Bcl-2: release of cytochrome c from mitochondria blocked. *Science* **275**: 1129–1132

Zha JP, Harada H, Yang E, Jockel J and Korsmeyer SJ (1996) Serine phosphorylation of death agonist BAD in response to survival factor results in binding to 14–3–3 not BCL-x(L). *Cell* **87**: 619–628

Zhang GJ, Kimijima I, Abe R, Watanabe T, Kanno M, Hara K and Tsuchiya A (1998) Apoptotic index correlates to bcl-2 and p53 protein expression, histological grade and prognosis in invasive breast cancers. *Anticancer Res* **18**: 1989–1998

 *© 1999 Cancer Research Campaign*
